# Mobile health technology for diverse populations: challenges and opportunities

**DOI:** 10.1038/s41746-021-00500-w

**Published:** 2021-09-06

**Authors:** James A. Diao, Joseph Kvedar

**Affiliations:** 1 Harvard Medical School, Boston, MA USA; 2 Mass General Brigham, Boston, MA USA

**Keywords:** Outcomes research, Hypertension

## Abstract

Nearly half of US adults have hypertension, and three in four cases are not well-controlled. Due to structural barriers, underserved communities face greater burdens of disease, less consistent management, and worse outcomes. Mobile technology presents an opportunity to reduce financial, geographic, and workforce barriers, but little data currently support its use in populations with digital disparities. A recent article by Khoong et al. systematically reviews the literature to quantify outcomes for these populations and provide a roadmap toward more inclusive mobile health strategies.

Nearly half of US adults have hypertension, and three in four cases are not well-controlled^1^. Due to structural barriers, underserved communities face greater burdens of disease, less consistent management, and worse outcomes^1^. Mobile technology presents an opportunity to reduce financial, geographic, and workforce barriers, but little data currently support its use in populations with digital disparities^2^. A recent article by Khoong et al^3^. systematically reviews the literature to quantify outcomes for these populations and provide a roadmap toward more inclusive mobile health strategies.

Khoong et al. searched through literature on mobile self-management of hypertension and identified 25 studies with sufficient numbers of non-White, elderly, or lower education participants. Of these, 15 were randomized trials. Reassuringly, all studies reporting engagement outcomes achieved >80% ratings on measures such as ease of use, usefulness, and satisfaction. Still, fewer than half observed significant improvements in systolic blood pressure (47%), medication adherence (25%), or blood pressure control (25%). A formal meta-analysis of outcomes at 6 months failed to demonstrate systolic blood pressure improvement relative to control groups (*P* = 0.48). Studies enriched in racial/ethnic minorities were the least likely to report significant improvement (17%), followed by those with lower educational attainment (56%) and increased age (67%).

These results indicate several challenges for the field. Few digital health studies collected information on race/ethnicity, digital literacy, or education, despite their considerable significance for outcomes and disparities. The relative dearth of studies in vulnerable populations indicates the need for improved representation and may explain why benefits noted in prior meta-analyses^4–6^ were not observed here. The study also points to several opportunities. Nearly half (44%) of included studies were conducted in 2019—the most recent year evaluated—and nearly all (92%) were conducted within 5 years, suggesting a shrinking data deficit. Khoong et al.’s data also reflect uniformly high interest and engagement, a critical first step for future equity efforts.

This review has limitations. The compared studies vary significantly by demographics, intervention design, and assessed quality, potentially confounding meta-analysis results. In addition, the inclusion criteria excluded studies in low-income countries and rural settings—both significant underserved populations—and grouped analyses may obscure intersectional or population-specific barriers. As the literature continues to expand, the more comprehensive and fine-grained assessment may become increasingly feasible. Future work should investigate specific features that enable successful intervention across populations and target gaps in digital inclusion^7^. In the meantime, clinical trials and user studies should standardize the collection of demographic and outcome data needed for rigorous validation of new technologies.

Mobile health promises to empower patients with their own health information, enable pattern recognition from longitudinal data, and ultimately, improve outcomes^8^. But as the technology gains traction, ongoing disparities prompt an important question: outcomes for whom? In their review, Khoong et al. offer much-needed data to quantify ongoing challenges and opportunities for deploying mobile health in diverse populations.
